# The effect of an intervention to promote isoniazid preventive therapy on leadership and management abilities

**DOI:** 10.5588/pha.24.0002

**Published:** 2024-06-01

**Authors:** C. Christian, E. Kakande, V. Nahurira, L.B Balzer, A. Owaraganise, J.R. Nugent, W. DiIeso, D. Rast, J. Kabami, J.J. Peretz, C.S. Camlin, S.B. Shade, M.R. Kamya, D.V. Havlir, G. Chamie

**Affiliations:** ^1^ University of California San Francisco, San Francisco, CA, USA;; ^2^ Infectious Diseases Research Collaboration, Uganda;; ^3^ University of California, Berkeley, CA,; ^4^ University of Massachusetts, Amherst, MA, USA;; ^5^ Sustainable East Africa Research Collaboration (SEARCH)-IPT Trial, Uganda,; ^6^ Makerere University, Kampala, Uganda

**Keywords:** health system strengthening, implementation science, tuberculosis, IPT, sub-Saharan Africa, Change Leadership Questionnaire, Leadership Behavior Description Questionnaire

## Abstract

**BACKGROUND:**

Across sub-Saharan Africa, mid-level healthcare managers oversee implementation of national guidelines. It remains unclear whether leadership and management training can improve population health outcomes.

**METHODS:**

We sought to evaluate leadership/management skills among district-level health managers in Uganda participating in the SEARCH-IPT randomised trial to promote isoniazid preventive therapy (IPT) for persons with HIV (PWH). The intervention, which led to higher IPT rates, included annual leadership/management training of managers. We conducted a cross-sectional survey assessing leadership/management skills among managers at trial completion. The survey evaluated self-reported use of leadership/management tools and general leadership/management. We conducted a survey among a sample of providers to understand the intervention’s impact. Targeted minimum loss-based estimation (TMLE) was used to compare responses between trial arms.

**RESULTS:**

Of 163 managers participating in the SEARCH-IPT trial, 119 (73%) completed the survey. Intervention managers reported more frequent use of leadership/management tools taught in the intervention curriculum than control managers (+3.64, 95% CI 1.98–5.30, *P* < 0.001). There were no significant differences in self-reported leadership skills in the intervention as compared to the control group. Among providers, the average reported quality of guidance and supervision was significantly higher in intervention vs control districts (+1.08, 95% CI 0.63–1.53, *P* = 0.001).

**CONCLUSIONS:**

A leadership and management training intervention increased the use of leadership/management tools among mid-level managers and resulted in higher perceived quality of supervision among providers in intervention vs control districts in Uganda. These findings suggest improved leadership/management among managers contributed to increased IPT use among PWH in the intervention districts of the SEARCH-IPT trial.

## Abstract (fr)

**CONTEXTE:**

Dans toute l'Afrique subsaharienne, les gestionnaires de soins de santé de niveau intermédiaire supervisent la mise en œuvre des directives nationales. Il n'est toujours pas clair si la formation en leadership et en gestion peut améliorer les résultats en matière de santé de la population.

**MÉTHODES:**

Nous avons cherché à évaluer les compétences en leadership et en gestion des responsables de la santé au niveau des districts en Ouganda participant à l'essai randomisé SEARCH-IPT visant à promouvoir le traitement préventif à l'isoniazide (TPI) pour les personnes vivant avec le VIH (PWH, pour l’anglais « *people living with HIV* »). L'intervention, qui a permis d'augmenter les taux de TPI, comprenait une formation annuelle en leadership et en gestion des gestionnaires. Nous avons mené une enquête transversale pour évaluer les compétences en leadership et en gestion des gestionnaires à la fin de l'essai. L'enquête a évalué l'utilisation autodéclarée d'outils de leadership et de gestion et de leadership et de gestion en général. Nous avons mené une enquête auprès d'un échantillon de prestataires pour comprendre l'impact de l'intervention. L'estimation ciblée basée sur les pertes minimales (TMLE, « *Targeted minimum loss-based estimation* ») a été utilisée pour comparer les réponses entre les groupes de l'essai.

**RÉSULTATS:**

Sur les 163 gestionnaires qui ont participé à l'essai SEARCH-IPT, 119 (73%) ont répondu au sondage. Les gestionnaires d'intervention ont déclaré utiliser plus fréquemment les outils de leadership/gestion enseignés dans le programme d'intervention que les gestionnaires de contrôle (+3,64 ; IC à 95% 1,98–5,30 ; *P* < 0,001). Il n'y avait pas de différences significatives dans les compétences de leadership autodéclarées dans l'intervention par rapport au groupe témoin. Parmi les prestataires, la qualité moyenne déclarée de l'orientation et de la supervision était significativement plus élevée dans les districts d'intervention que dans les districts témoins (+1,08 ; IC à 95% 0,63–1,53 ; *P* = 0,001).

**CONCLUSIONS:**

Une intervention de formation au leadership et à la gestion a permis d'accroître l'utilisation d'outils de leadership et de gestion parmi les cadres intermédiaires et d'améliorer la perception de la qualité de la supervision parmi les prestataires dans les districts d'intervention par rapport aux districts de contrôle en Ouganda. Ces résultats suggèrent que l'amélioration du leadership et de la gestion chez les gestionnaires a contribué à l'augmentation de l'utilisation du TPI chez les personnes handicapées dans les districts d'intervention de l'essai SEARCH-IPT.

Across sub-Saharan Africa (SSA), healthcare “middle managers” oversee the implementation of national guidelines and play a critical role in the functioning of the health system. As sub-national leaders and managers, they operate at the nexus of guidelines and implementation, setting healthcare priorities, managing frontline providers, and overseeing budgetary, education, and operational aspects of healthcare service delivery. Despite these responsibilities, mid-level healthcare managers are often clinicians with little or no formal leadership and management training. Leadership and management have been identified by the WHO and the Lancet Commission on Health Systems as key areas of interest in health systems strengthening and governance.^1,2^ Lack of training in this area may limit effectiveness of mid-level managers, given that effective leadership skills have been associated with positive health outcomes.

While several studies have explored interventions to enhance leadership abilities among healthcare professionals in SSA, only a few have assessed the impact of leadership training interventions on health outcomes. A recent scoping review of interventions aimed at strengthening leadership capabilities of health professionals in SSA found that 13 of 27 studies identified also included management training, but only 4 of 27 (15%) evaluated health or health systems outcomes,^3^ including improvements in hospital performance standards,^4^ and health services coverage.^5^ Similarly, only 7 of 27 (26%) studies in the review evaluated learning of leadership/management skills. Given the role mid-level managers play in healthcare service delivery, further evaluation of interventions aimed at improving leadership/management skills of this cadre of managers is needed.

In the SEARCH-IPT trial, a multi-component intervention among mid-level managers in Uganda resulted in an increased uptake of isoniazid preventive therapy (IPT) for persons with HIV (PWH) compared to standard care, after considering a 100-day push led by the Ministry of Health in Uganda and the U.S. President’s Emergency Plan for AIDS Relief. One component of the intervention was annual leadership/management training provided to mid-level managers by business professionals over 3 years, as previously described.^6^ The impact of changes in leadership and management on the outcomes of the SEARCH-IPT trial remains unclear.

We sought to evaluate self-reported leadership and management skills among mid-level managers who participated in the SEARCH-IPT trial. We also sought to identify perceptions of frontline providers on the quality of the supervision of these mid-level managers to better understand how the SEARCH-IPT trial’s intervention led to changes in IPT initiation among PWH.

## METHODS

### Study design

We conducted a cross-sectional survey assessing leadership and management skills among district-level managers in Uganda at the end of the SEARCH-IPT trial. The SEARCH-IPT trial is a cluster-randomised trial that evaluated the effect of a multi-component intervention among district-level health managers on IPT initiation rates for PWH and in care in three regions of Uganda. We have previously published the trial’s methods and results.^6^ In brief, the trial enrolled district health officers, the highest-ranking Ministry of Health leaders in each district, and TB supervisors who oversee TB-specific activities and report to health officers. Each district in Uganda has one district health officer and one TB supervisor. We created 14 pair-matched groups of these health managers (with 4–7 managers per group) and randomised the groups 1:1 to the SEARCH-IPT intervention or control.

As a key component of the SEARCH-IPT intervention, we offered intensive interactive leadership/management training courses at annual “mini-collaborative” meetings over 3 years. Each course was designed and led by two international business consultants, with courses emphasising tools to improve leadership/management skills adapted to a Ugandan context. The three courses in this “Mini-MBA” curriculum focused on 1) Kotter’s 8-Step Model for Change, 2) Objective Key Results (OKRs), 3) and the Start/Stop/Continue Retrospective technique.^7–9^

In the present study, we sought to compare leadership and management skills by arm in an end-of-study survey among managers participating in the SEARCH-IPT trial. The survey evaluated the use of tools covered by the “Mini-MBA” curriculum, as well as general leadership and management capabilities using two established questionnaires from the leadership/management literature: the Change Leadership Questionnaire and the Leadership Behavior Description Questionnaire.^10,11^ We collected survey data from September 2021 to February 2022.

To understand the impact of the intervention on healthcare workers supervised by managers participating in the trial, we also conducted a survey among a sample of frontline providers after the first year of the SEARCH-IPT trial, between February and August 2019. We surveyed a convenience sample of frontline providers from clinics in intervention and control districts in the Southwest and East regions of the trial, based on proximity to study offices and quality of road to the clinics. At each clinic, we invited frontline providers to participate in the study, including nurses, clinical officers, counsellors, peer educators, midwives, and lab technicians. We surveyed frontline providers from eight districts in total (four control and four intervention districts).

### Outcomes and measures

#### Mini-MBA curriculum questionnaire

We asked five questions about the frequency of use of skills taught during the ‘Mini-MBA’ curriculum using a Likert scale that ranged from 1 = rarely to 4 = always, and calculated an aggregate score of the five questions for each respondent.

#### Change Leadership Questionnaire

The Change Leadership Questionnaire is a survey designed for self-assessment of leadership skills in five areas representing leadership qualities: visionary, inspirer, supporter, problem solver, and change manager. We created a modified version of the questionnaire, shortened to reduce completion time (Supplementary Data), and calculated an aggregate score for each area.

#### Leadership Behavior Description Questionnaire

The LBDQ is a 100-item questionnaire designed to measure self-perceived leadership/management abilities.^12^ Each question describes a behaviour and asks the participant to rank how often they engage in that behaviour, from ‘Always’ to ‘Never’. The LBDQ is divided into 12 subcategories, each of which receives a score ranging from a maximum of 25 or 50, depending on the category, with higher scores representing high self-assessed abilities and lower scores representing low abilities in that area.^11^

#### Frontline Provider Survey

The survey evaluated frontline provider perceptions of the quality of guidance and supervision received from both district health officers and TB-specific managers during the trial. Responses to this survey question were scored using a 5-point Likert scale, with scores ranging from 1 (indicating very low quality) to 5 (indicating very high quality).

### Statistical analysis

We used targeted minimum loss-based estimation (TMLE) to compare survey responses between trial arms, accounting for the clustering of the districts in the trial design and adjusting for missingness.^13,14^ For each of the survey questions and for survey sub-domains, as applicable, we evaluated the difference in average responses between intervention and control groups. For the specific content from the ‘Mini-MBA’, we compared scores for each question individually and an overall score, calculated by summing the scores of the five questions. Likewise, for the Change Leadership Questionnaire, we compared scores for each sub-domain and an overall score, again calculated by summing all scores. For the LBDQ, we compared the scores of the 12 sub-domains as outlined by the published survey.^11^ For the frontline provider survey, TMLE was also used to evaluate the difference in the average rating of the manager’s quality of guidance and supervision by trial arm. All analyses were prespecified, and hypothesis testing was conducted using a two-sided test at the 5% significance level.

### Ethics approval and consent to participate

Approval was obtained by the Institutional Review Boards at the School of Medicine Research and Ethics Committee at Makerere University School of Medicine, Kampala, Uganda (2017-116), the University of California, San Francisco, San Francisco, CA, USA (17-22136), and the Uganda National Council for Science and Technology, Kampala, Uganda (HS 2331). Written, informed consent was obtained by all study participants prior to study activities.

## RESULTS

### Study population

Of 163 managers participating in the SEARCH-IPT trial, 119 (73%) completed leadership and management survey, with at least one manager surveyed from 78 (94%) of the 83 districts in the trial. Forty-eight (40%) survey respondents were lead managers, whereas 71 (60%) were TB-specific managers (Table 1).

**TABLE 1.** Characteristics of the districts involved in the SEARCH-IPT trial in Uganda and the participants who completed Leadership and Management Survey at the end of Phase I.

	Intervention Group	Control Group
	*n* (%)	*n* (%)
**A)** Characteristics of districts in the SEARCH-IPT Trial (*n* = 15)		
Clusters, *n*	7	7
Districts, *n*	43	39
Regions, *n*		
Southwest	13	12
East	12	11
East-Central	18	16
Districts per cluster, median [IQR]	5 [5–6]	5 [5–6]
**B)** Characteristics of participants completing leadership and management survey		
Mid-level managers enrolled in trial, *n*	86	77
Cadre of managers responding to survey		
Lead manager	32 (45)	16 (33)
TB-specific manager	39 (55)	32 (67)
Total	71	48
Sex of survey respondents		
Male	63 (89)	42 (87.5)
Female	8 (11)	6 (12.5)
Region of survey respondents		
Southwest	26 (37)	21 (44)
East	16 (23)	14 (29)
East-Central	29 (41)	13 (27)

*District mini-collaboratives (intervention groups) or mini-groups (control group).

IQR = interquartile range.

### Questionnaires

#### Mini-MBA content

When questioned about the content covered in the 'Mini-MBA' curriculum, it was found that the intervention group exhibited higher average overall survey scores than the control group (+3.64, 95% CI 1.98–5.30; *P* < 0.001), suggesting that the intervention group demonstrated a more frequent utilisation of leadership and management skills taught as part of the SEARCH-IPT intervention than the control group (Table 2). When evaluating specific skills used, intervention group managers reported more frequent use of Kotter’s 8-Step Model for Change (Change +1.09, 95% CI 0.68–1.50; *P* < 0.001), communication of their change vision (+1.19, 95% CI 0.67–1.72; *P* < 0.001), and use of “Start, Stop, Continue” team feedback (+0.79, 95% CI 0.23–1.35; *P* = 0.007), compared to control group managers.

**TABLE 2.** Average scores on survey assessing use of skills taught as part of the SEARCH-IPT intervention (i.e., the “Mini-MBA” curriculum) among mid-level managers intervention and control participating in the SEARCH-IPT trial in Uganda (*n =* 119 survey respondents).

‘Mini-MBA’ Content Questions	Intervention Group scores	Control Group scores	Intervention Group vs. Control Group Difference (95% CI)	*P*-value
	Mean (95% CI)	Mean (95% CI)		
Overall ‘Mini-MBA’ content	14.18 (13.31 to 15.05)	10.54 (9.12 to 11.96)	3.64 (1.98 to 5.30)	<0.001
Kotter’s 8-Step Method: “I use Kotter’s 8-Step Model to create a change vision for key performance indicators in my district”	2.46 (2.32 to 2.60)	1.37 (0.99 to 1.76)	1.09 (0.68 to 1.50)	<0.001
Communicates change vision: “Using Kotter’s principles, I communicate and emphasise the change I want to see at every opportunity”	2.65 (2.35 to 2.96)	1.46 (1.03 to 1.88)	1.19 (0.67 to 1.72)	<0.001
Short-term wins: “I celebrate short-term wins or improvements in performance with my team to motivate them”	3.24 (2.99 to 3.50)	2.86 (2.55 to 3.17)	0.38 (‒0.02 to 0.78)	0.062
Objective Key Results: “I use ‘Objectives and Key Results (OKRs)’ to set goals and measure progress in my district”	3.19 (3.09 to 3.29)	2.92 (2.56 to 3.28)	0.27 (‒0.10 to 0.65)	0.146
Start, Stop, Continue: “I use the ‘Start, Stop, Continue’ model to evaluate what strategies do and do not work in my district every quarter”	2.62 (2.30 to 2.95)	1.84 (1.38 to 2.29)	0.79 (0.23 to 1.35)	0.007

CI = confidence interval.

#### Change Leadership Questionnaire

The overall change leadership score across the five areas assessed did not show significant differences between intervention and control managers (Table 3).

**TABLE 3.** Results of the modified Change Leadership Questionnaire between the mid-level managers intervention and control participating in the SEARCH-IPT study in Uganda (*n =* 119).

Subscale domains	Intervention Group scores	Control Group scores	Intervention Group vs. Control Group Difference (95% CI)	*P*-value
	Mean (95% CI)	Mean (95% CI)		
Overall change leadership	83.19 (80.56 to 85.81)	84.24 (81.36 to 87.12)	−1.05 (−4.95 to 2.85)	0.589
Visionary	17.26 (16.90 to 17.61)	17.46 (16.94 to 17.97)	−0.20 (−0.83 to 0.43)	0.526
Inspirer	16.34 (15.75 to 16.93)	16.84 (16.19 to 17.48)	−0.50 (−1.37 to 0.38)	0.257
Supporter	16.70 (16.04 to 17.35)	16.66 (16.03 to 17.29)	0.03 (−0.88 to 0.95)	0.939
Problem solver	16.08 (15.19 to 16.97)	16.40 (15.65 to 17.15)	−0.32 (−1.48 to 0.84)	0.583
Change manager	16.82 (16.29 to 17.34)	16.80 (15.89 to 17.71)	0.02 (−1.03 to 1.06)	0.975

CI = confidence interval.

#### Leadership Behavior Description Questionnaire

For each domain, the intervention group did not show significantly higher self-reported leadership skills as compared to control (Table 4). For the sub-domain ‘Tolerance and Uncertainty’, the control group reported higher skills in this area compared to intervention (difference = –1.9, 95% CI –3.5 to –0.3; *P* = 0.024).

**TABLE 4.** Results of the Leadership Behavior Description Questionnaire conducted among mid-level managers in the intervention and control arms of the SEARCH-IPT trial in Uganda (*n =* 119).

Subscale domains	Intervention Group scores	Control Group scores	Intervention Group vs. Control Group Difference (95% CI)	*P*-value
	Mean (95% CI)	Mean (95% CI)		
Representation* (‘speaks and acts as the representative of the group’)^†^	21.0 (20.5 to 21.5)	21.0 (20.1 to 21.9)	0.0 (−1.1 to 1.0)	0.979
Demand reconciliation* (‘reconciles conflicting demands and reduces disorder to system’)^†^	18.8 (18.3 to 19.2)	19.3 (18.4 to 20.2)	−0.5 (−1.5 to 0.5)	0.303
Tolerance of uncertainty (‘is able to tolerate uncertainty and postponement without anxiety or upset’)^†^	32.3 (31.6 to 33.0)	34.1 (32.7 to 35.6)	−1.9 (−3.5 to −0.3)	0.024
Persuasiveness (‘uses persuasion and argument effectively; exhibits strong convictions’)^†^	38.5 (37.9 to 39.0)	39.9 (38.4 to 41.3)	−1.4 (−3.0 to 0.2)	0.076
Initiation of structure (‘clearly defines own role, and lets followers know what is expected’)^†^	43.2 (42.6 to 43.9)	43.9 (42.7 to 45.0)	−0.6 (−2.0 to 0.7)	0.329
Tolerance and freedom (‘allows followers to scope for initiative, decision and action’)^†^	37.9 (37.2 to 38.6)	38.7 (37.1 to 40.3)	−0.8 (−2.6 to 0.9)	0.341
Role assumption (‘actively exercises the leadership role rather than surrendering leadership to others’)^†^	39.3 (38.4 to 40.2)	40.1 (38.9 to 41.2)	−0.8 (−2.2 to 0.7)	0.299
Consideration (‘regards the comfort, well-being, status, and contributions of followers’)^†^	40.1 (39.5 to 40.6)	40.8 (39.5 to 42.1)	−0.8 (−2.2 to 0.6)	0.281
Production emphasis (‘applies pressure for productive output’)^†^	40.4 (39.7 to 41.1)	41.5 (40.4 to 42.6)	−1.1 (−2.4 to 0.3)	0.109
Predictive accuracy* (‘exhibits foresight and ability to predict outcome accurately’)^†^	19.0 (18.6 to 19.4)	18.9 (18.0 to 19.7)	0.2 (−0.7 to 1.1)	0.713
Integration* (‘maintains a closely knit organisation; resolves inter-member conflicts’)^†^	22.9 (22.4 to 23.4)	23.6 (23.0 to 24.1)	−0.6 (−1.3 to 0.1)	0.089
Superior orientation (‘maintains cordial relations with superiors; has influence with them; is striving for higher status’)^†^	41.4 (40.5 to 42.2)	41.6 (40.3 to 42.9)	−0.3 (−1.8 to 1.3)	0.741

* Scores in these sub-scales comprised of five questions for a maximum of 25 points. All other sub-scales are out of 50 points.

† Definitions of the sub-scales are from the Leadership Behavior Description Questionnaire manual.^11^

CI = confidence interval.

#### Frontline providers

One-year after trial intervention initiation, 54 frontline providers completed the survey. Providers in intervention group districts reported an average score for the quality of guidance and supervision by their district-level managers of 4.19 (95% CI 3.69–4.63). This score ranged between 'high quality' and 'very high quality' guidance and supervision. In contrast, providers in control group districts reported an average score of 3.08 (95% CI 2.29–3.87), indicating “moderate quality” guidance and supervision. Overall, frontline providers from intervention districts reported a significantly higher average score for the quality of guidance and supervision compared to those from control districts (with an average score 1.08 units higher, 95% CI 0.63–1.53; *P* = 0.001).

## DISCUSSION

In a cross-sectional evaluation of mid-level health managers across Uganda who participated in the SEARCH-IPT trial to increase IPT initiation for PWH, managers reported more frequent use of leadership and management tools in intervention than control group districts. Frontline providers reported a higher quality of guidance and supervision from managers in intervention compared to control group districts. As previously described, the SEARCH-IPT trial's multi-component intervention resulted in increased IPT knowledge and collaboration among managers. It led to significantly increased IPT initiation rates after accounting for large increases in IPT from a national “100-day IPT push,” in Uganda during the trial.^6^ The post-trial survey findings of greater use of specific leadership/management tools by managers and higher quality supervision perceived by providers in intervention districts, suggest that improvements in leadership and management contributed to the intervention effects found in the SEARCH-IPT trial.

The increased use of specific leadership and management skills from the ‘Mini-MBA’ training by the intervention group suggests that the training curriculum provided practical and actionable tools that intervention managers applied in their districts. Interestingly, control group managers also reported using OKRs as a leadership/management tool at a high frequency, albeit less frequently than intervention managers. The provision of additional leadership/management tools, such as Kotter’s 8-Step Model for Change and the Start/Stop/Continue Retrospective technique, may have expanded the “menu” of tools available to intervention managers and allowed for greater flexibility in their role as leaders and managers. Whereas OKRs and Start/Stop/Continue Retrospective techniques are designed to be simple and easy to use, Kotter’s 8-Step Model for Change is relatively more complex and explicitly geared toward implementing changes within organisations in a lasting, sustainable way.^7^ Taken together, these findings indicate that although there was some familiarity with leadership/management techniques among both control and intervention managers, our ‘Mini-MBA’ curriculum adapted to a Ugandan context, led to higher reported use of specific leadership/management tools. These tools were acceptable and usable by intervention managers and may have contributed to the higher quality of supervision reported by frontline providers in intervention districts, as well as the intervention effects in the SEARCH-IPT trial.

In contrast, when using previously established leadership and management questionnaires (the LBDQ and Change Leadership Survey), we did not find significant differences in self-assessed leadership or management skills, apart from one sub-domain in the LBDQ (the Tolerance of Uncertainty sub-domain) in which control group managers reported higher competency than intervention managers. To date, although the LBDQ has been used to evaluate leadership skills across a wide variety of fields (i.e. business,^15^ education,^16^ military,^17^ etc.), its use to evaluate health care providers and managers has been limited. For example, in one study the LBDQ was used to evaluate leadership among cardiopulmonary resuscitation teams, and found an association between enhanced communication and collaboration among resuscitation teams and patient outcomes.^18^ One potential explanation for the lack of difference between trial arms in these established questionnaires, despite the leadership and management curriculum provided to intervention group managers, is over-confidence in skills and abilities among control group managers. Specifically, the Mini-MBA curriculum may have raised awareness of intervention group managers’ areas for improvement or the broader scope of available leadership/management skills and techniques, resulting in relatively lower self-reported skills than control group managers: a finding consistent with the Dunning-Kruger effect.^19^ As such, the intervention group managers may have underestimated their skills, and/or the control group managers may have overestimated their skills, resulting in the lack of significant differences observed.

Despite the lack of differences observed in the LBDQ and Change Leadership Survey, frontline providers overseen by intervention managers reported a higher quality of guidance and supervision compared to providers overseen by control group managers. Studies have found associations between improved leadership and management among supervisors and improved outcomes at the clinic level and more efficient use of resources.^20,21^ In a study based in Kenya and South Africa, the relationship between managers and frontline providers at the sub-national level was an important factor in the success of accountability mechanisms and reaching health outcome targets at the clinic level.^22^ Our finding that frontline providers in intervention districts noted higher quality of guidance and supervision from their managers reinforces our finding of higher reported use of the leadership/management tools among intervention group managers. Though limited to a sample of frontline providers from intervention and control districts, this finding suggests that the leadership and management training contributed to the higher IPT initiation rate in intervention vs control districts in the SEARCH-IPT trial.^6^

This study has limitations. First, our assessment of leadership/management skills in the end-of-study survey relied on self-report and has the potential for reporting bias due to over-inflation of skills, which may have biased our results to a null effect. To address this limitation, leadership/management assessments were supported by survey data collected from frontline providers. Second, we collected survey data cross-sectionally, preventing evaluation of change in leadership/management skills over time. However, any differences at baseline are likely to have been small, with mid-level managers having comparable baseline leadership/management skills, given the randomised study design of the SEARCH-IPT trial.

## CONCLUSIONS

Leadership and management training provided as part of an intervention for mid-level managers in Uganda was associated with greater reported use of leadership/management tools in intervention compared to control districts and resulted in higher perceived quality of supervision among frontline providers in intervention vs control districts. These findings suggest that improved leadership/management among managers contributed to intervention effectiveness in increasing IPT use among PWH in the SEARCH-IPT cluster-randomised trial.

## Supplementary Material


