# Supplementary material for: The effect of an intervention to promote isoniazid preventive therapy on leadership and management abilities
Source: Public Health Action. 2024 Jun 1;14(2):56–60. doi: 10.5588/pha.24.0002 (PMC11216294; doi:10.5588/pha.24.0002)
Supplement: Supplementary file 1 [file iutld_pha_24.0002_supplementarydata1.pdf]

http://dx.doi.org/10.5588/pha.24.0002

SUPPLEMENTARY DATA

The effect of an intervention to promote isoniazid preventive therapy on leadership and management abilities

Change Leadership Questionnaire - *Modified from Gilley (2005) (1)*

Skill Area: Visionary

1. I have a “leader vision” of health services in my district, including its potential and capabilities.

   ☐ 1. Rarely

   ☐ 2. Sometimes

   ☐ 3. Frequently

   ☐ 4. Always

2. I am creative, able to envision that which does not yet exist.

   ☐ 1. Rarely

   ☐ 2. Sometimes

   ☐ 3. Frequently

   ☐ 4. Always

3. I draw people in and build networks of people working toward a common goal.

   ☐ 1. Rarely

   ☐ 2. Sometimes

   ☐ 3. Frequently

☐ 4. Always

4. I am confident in my ability to lead others.

   ☐ 1. Rarely

   ☐ 2. Sometimes

   ☐ 3. Frequently

   ☐ 4. Always

5. I willingly and enthusiastically share the purpose and vision of the health department of my district to ensure that the health workers I supervise “own the vision”.

   ☐ 1. Rarely

   ☐ 2. Sometimes

   ☐ 3. Frequently

   ☐ 4. Always

Skill Area: Inspirer

1. I allow health workers in my district to participate in the development of the district’s health vision.

   ☐ 1. Rarely

   ☐ 2. Sometimes

   ☐ 3. Frequently

   ☐ 4. Always

2. I work collaboratively with health workers in my district to accomplish the district’s health goals and objectives.

- 1. Rarely
- 2. Sometimes
- 3. Frequently
- 4. Always

3. I am the first to incorporate new ways and change into my daily routine.
    - 1. Rarely
    - 2. Sometimes
    - 3. Frequently
    - 4. Always
4. I help people to see “what’s in it for them” with regard to impending change.
    - 1. Rarely
    - 2. Sometimes
    - 3. Frequently
    - 4. Always
5. My behaviors are consistent with my words.
    - 1. Rarely
    - 2. Sometimes
    - 3. Frequently
    - 4. Always

Skill Area: Supporter

1. I identify and eliminate barriers to change within my district.

    ☐ 1. Rarely

    ☐ 2. Sometimes

    ☐ 3. Frequently

    ☐ 4. Always

2. I encourage people to share their opinions, concerns, and suggestions for improvement.

    ☐ 1. Rarely

    ☐ 2. Sometimes

    ☐ 3. Frequently

    ☐ 4. Always

3. I am an advocate for my health workers and pursue needed resources aggressively.

    ☐ 1. Rarely

    ☐ 2. Sometimes

    ☐ 3. Frequently

    ☐ 4. Always

4. I develop a solid communication pattern with health workers in my district in terms of frequency and depth.

    ☐ 1. Rarely

    ☐ 2. Sometimes

    ☐ 3. Frequently

☐ 4. Always

5. I hold myself accountable for the performance of my health workers and the success of our projects.

   ☐ 1. Rarely

   ☐ 2. Sometimes

   ☐ 3. Frequently

   ☐ 4. Always

Skill Area: Solver

1. I am able to gather data, draw conclusions, propose and assess alternatives, and recommend viable solutions.

   ☐ 1. Rarely

   ☐ 2. Sometimes

   ☐ 3. Frequently

   ☐ 4. Always

2. I demonstrate resourcefulness in my approaches to new and existing problems or opportunities.

   ☐ 1. Rarely

   ☐ 2. Sometimes

   ☐ 3. Frequently

   ☐ 4. Always

3. I work collaboratively with health workers to evaluate the status of change efforts and modify as needed.

- ☐ 1. Rarely
- ☐ 2. Sometimes
- ☐ 3. Frequently
- ☐ 4. Always

4. I think “outside the box” and encourage the same in others.

    - ☐ 1. Rarely
    - ☐ 2. Sometimes
    - ☐ 3. Frequently
    - ☐ 4. Always

5. I think broadly to generate alternatives and engage in thorough analysis of what strategies are possible to implement.

    - ☐ 1. Rarely
    - ☐ 2. Sometimes
    - ☐ 3. Frequently
    - ☐ 4. Always

Skill Area: Change Manager

1. I understand the immense complexities of change, including planning, implementation, and human reactions.

    - ☐ 1. Rarely
    - ☐ 2. Sometimes
    - ☐ 3. Frequently

- 4. Always

2. I openly communicate with health workers to meet their needs and help them work through change.

    - 1. Rarely
    - 2. Sometimes
    - 3. Frequently
    - 4. Always

3. I work with health workers to collaboratively set realistic, challenging, yet attainable goals and expectations.

    - 1. Rarely
    - 2. Sometimes
    - 3. Frequently
    - 4. Always

4. I understand that resistance to change is natural yet can be overcome.

    - 1. Rarely
    - 2. Sometimes
    - 3. Frequently
    - 4. Always

5. I understand the importance of goal setting and its relationship to health worker motivation.

    - 1. Rarely

- ☐ 2. Sometimes
- ☐ 3. Frequently
- ☐ 4. Always

References

1. Gilley AM. The manager as change leader. Westport, Conn: Praeger Publishers; 2005.
